# Ocular toxicity of investigational anti-cancer drugs in early phase clinical trials

**DOI:** 10.1007/s10637-022-01321-8

**Published:** 2022-12-05

**Authors:** Shigemasa Takamizawa, Yuki Katsuya, Yi-Ning Chen, Takaaki Mizuno, Takafumi Koyama, Kazuki Sudo, Tatsuya Yoshida, Shunsuke Kondo, Satoru Iwasa, Kan Yonemori, Toshio Shimizu, Noboru Yamamoto, Shigenobu Suzuki

**Affiliations:** 1 Department of Experimental Therapeutics, National Cancer Center Hospital, 5-1-1 Tsukiji, Chuo-Ku, Tokyo, 104-0045 Japan; 2 Department of Ophthalmic Oncology, National Cancer Center Hospital, Tokyo, Japan

**Keywords:** Adverse event, Anti-cancer drug, Clinical trial, Early phase, Ocular toxicity

## Abstract

Ocular toxicities arising from anti-cancer drugs occur sporadically and are sometimes underestimated because they are not life-threatening. Reports focusing on ocular toxicities from cancer therapy are limited. We investigated the detailed progress of ocular toxicities of anti-cancer drugs including first-in-class ones. A retrospective review of medical records was conducted for patients who were involved in early phase clinical trials with scheduled ophthalmologic examinations according to their protocols between January 2014 and August 2021. Patients with ocular toxicity suspected to be related to the investigational drugs in the ophthalmic examination were investigated in detail. In total, 37 ocular toxicities related to investigational drugs occurred in 7.6% of patients (33/434). The median age of the 33 patients was 61 years (range, 33–76 years), and 20 were male. Causal drugs with a high incidence of ocular toxicities were HSP90 inhibitors and FGFR inhibitors. Retinopathy was most frequent, while conjunctivitis, dry eye, keratitis, keratopathy, and uveitis were also observed. Dim vision as a subjective finding was a unique adverse event. Most patients developed ocular toxicities even though their dose was below the drug’s maximum tolerated dose. Except for one case, all ocular toxicities occurred bilaterally. About 60% (22/37) of ocular toxicity cases needed a temporary hold of the drug. All except for three cases fully recovered. This study reported the risks and timing of the onset of a variety of ocular toxicities of anti-cancer drugs, which were fundamentally controllable. (Trial registration number. Retrospectively registered)

## Introduction

Anti-cancer therapy has made remarkable progress in recent years with the emergence of molecular targeted agents, immune checkpoint inhibitors (ICIs), and antibodies (monoclonal antibodies, antibody–drug conjugates [ADCs], and bispecific antibodies). Ocular toxicities arising from cancer therapy are observed sporadically. Known frequent ocular toxicities include lacrimal drainage obstruction caused by S-1, corneal disorders caused by cytarabine and S-1, and retinal detachment caused by mitogen-activated protein kinase (MEK)-inhibitors, and other ocular toxicities caused by ICIs, fibroblast growth factor receptor (FGFR) inhibitors, anti-human epidermal growth factor receptor 2 (HER2) monoclonal antibodies, and some ADCs [1, 2].

Since visual dysfunction is directly related to patients’ quality of life, careful management of ocular toxicities is necessary. However, ocular toxicity tends to be underestimated because it occurs at low frequency and is non-fatal. We investigated the detailed progression of ocular toxicities arising due to anti-cancer drugs in early phase clinical trials.

## Patients and methods

### Study cohort

We retrospectively reviewed the medical records of patients who participated in early phase clinical trials at the National Cancer Center Hospital (NCCH) (Tokyo, Japan) between January 2014 and August 2021. Early phase clinical trials which required an ophthalmologic examination according to their protocols before the start (for screening) and after completion (to confirm adverse events) of the administration of investigational drugs were included.

### Analysis item

We retrospectively reviewed patients’ medical records for details on sex, age (at enrolment), cancer type, details of the investigational drug (dose, date of initiation, and date of last dose), and ocular toxicity (symptoms, grade, association with the investigational drug, onset date, and recovery or last follow-up date). Symptoms and grades of ocular toxicity were assessed according to the Common Terminology Criteria for Adverse Events (version 5.0) [3].

This study was approved by the Institutional Review Board of NCCH (NCCH 2014–148), which waived the requirement for informed consent. The study was conducted according to the principles of the Declaration of Helsinki.

## Results

### Patient characteristics

Between January 2014 and August 2021, 42 studies requiring an ophthalmic examination were conducted, with a total of 434 participating patients. Of these, 37 ocular toxicities among 33 patients (7.6%) in 13 trials were judged to be related to the investigational drugs. The median age of the 33 patients with ocular toxicity was 61 years (range, 33–76 years). Twenty patients were male. The characteristics of the patients are shown in Table 1.

**Table 1:** Patient characteristics

Patients with ocular toxicity		n = 33	
Sex	Male (%)	20 (60.6)	
	Female (%)	13 (39.4)	
Age median (range), years		61 (33–76)	
Cancer type	Lung (%)	8 (24.2)	
	Hepatobiliary and pancreatic (%)	8 (24.2)	
	Gynecologic (%)	4 (12.1)	
	Gastrointestinal (%)	3 (9.1)	
	Urinary (%)	3 (9.1)	
	Breast (%)	2 (6.1)	
	Other (%)	5 (15.2)	
Agent		Patients with ocular toxicity	Total patients treated with investigational drugs
	AKT inhibitor + FGFR inhibitor	1	5
	Anti-PD-1 antibody	2	9
	AURORA inhibitor	2	13
	Axl/Mer inhibitor	1	11
	CLK inhibitor	2	22
	EP4 inhibitor	2*	31
	FGFR inhibitor	14*	26
	HER2-directed ADC	2	37
	HSP90 inhibitor	7	12
	RET inhibitor	1	2

*ADC* Antibody–drug conjugate, *CLK* CDC-like kinase, *EP4* prostaglandin E2 receptor 4, *FGFR* fibroblast growth factor receptor, *HER2* human epidermal growth factor receptor 2, *HSP90* heat shock protein 90, *PD-1* programmed death-1, *RET* rearranged during transfection

^*^One patient participated in both trials for an EP4 inhibitor and FGFR inhibitor

### Investigational drugs that induce ocular toxicity

**Fig. 1:**
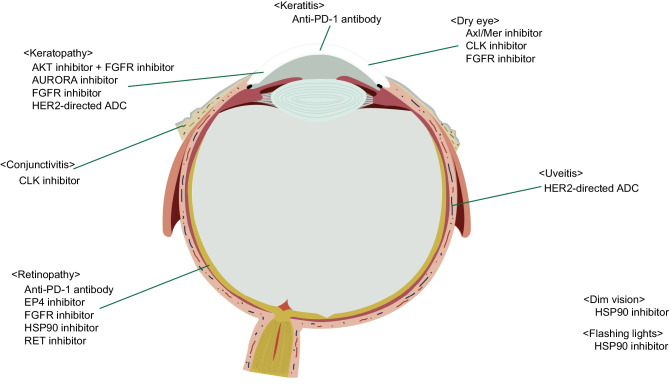
Investigational anti-cancer drugs per each ocular toxicity. Anatomical drawing of the eye and details of investigational drugs for each ocular toxicity

Drugs found to induce ocular toxicity were an AKT inhibitor plus FGFR inhibitor, anti-programmed death-1 (PD-1) antibody, AURORA inhibitor, Axl/Mer inhibitor, CDC-like kinase (CLK) inhibitor, prostaglandin E2 receptor 4 (EP4) inhibitor, FGFR inhibitor, HER2-directed ADC, heat shock protein 90 (HSP90) inhibitor, and rearranged during transfection (RET) inhibitor (Fig. 1). Drugs linked to a high incidence of ocular toxicities were HSP90 inhibitors and FGFR inhibitors, with any ocular toxicity appearing in 7 (58.3%) of the 12 patients treated with HSP90 inhibitors and 14 (53.8%) of the 26 patients treated with FGFR inhibitors (Tables 1 and 2).

**Fig. 2:**
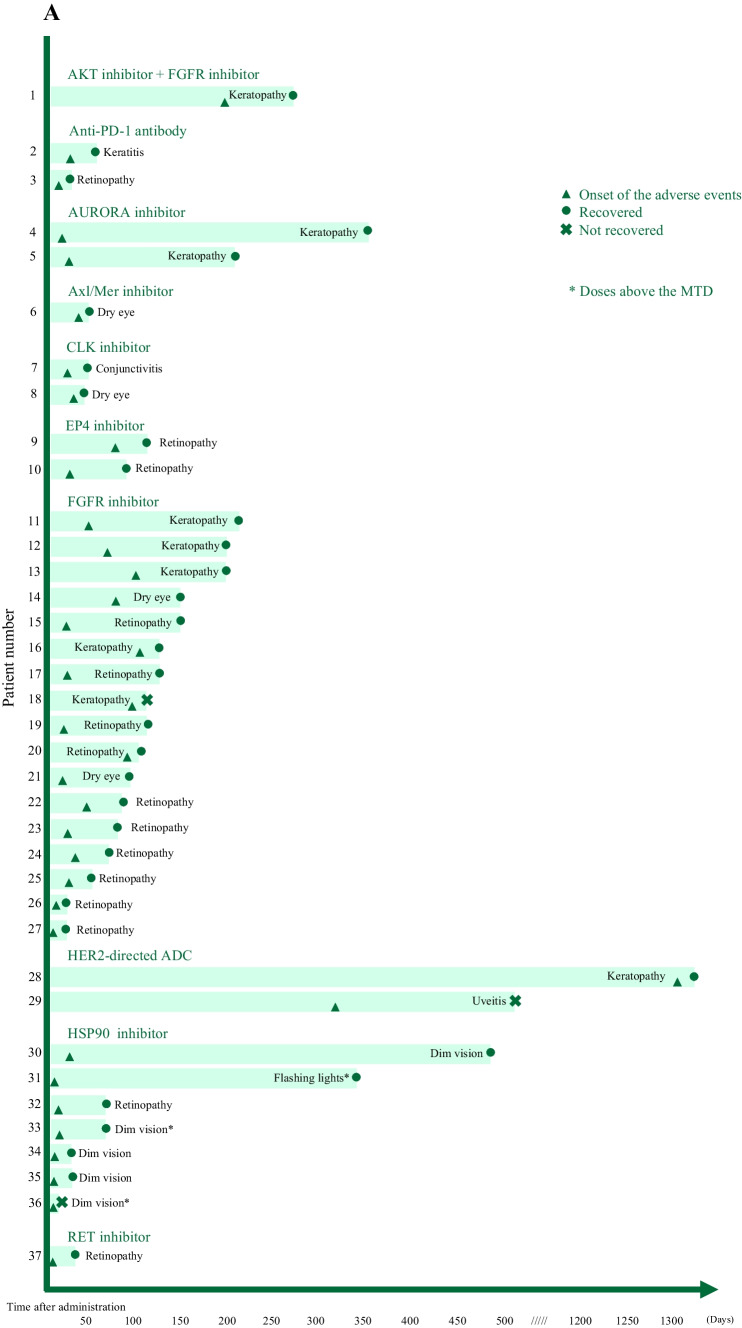

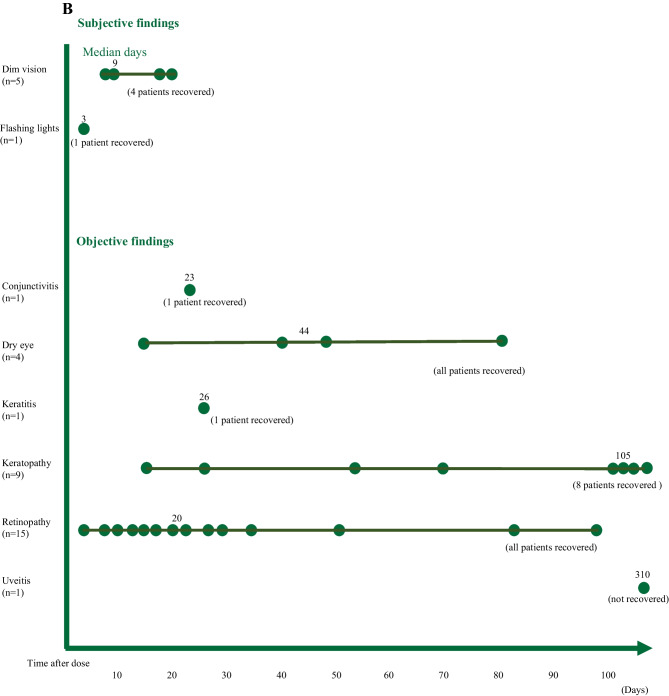
**A** Ocular toxicity by agent. Details of ocular toxicity (symptom, onset, and recovery) for each investigational drug. **B** Ocular toxicity by symptom. Days from cycle one to the onset of toxicity for each symptom

Ocular toxicity occurred within the first 50 days from the administration of drugs in 25 cases. In some cases, however, as in HER2-directed ADC, toxicity occurred more than 1000 days after initial administration of the drug, while the patient was still receiving the treatment (Fig. 2A). Only three patients were treated with doses that were above the drug’s maximum tolerated dose (MTD), while the other patients developed ocular toxicities despite receiving doses below the MTD.

### Symptoms of ocular toxicity

Ocular toxicities of most cases were mild, at grade 1–2. Grade 3 toxicity was observed in only two patients: keratopathy due to an FGFR inhibitor and flashing lights due to an HSP90 inhibitor (Table 2). Keratopathy showed varied timing of onset (14–1297 days from administration), with a case even occurring more than 1000 days after administration of the drug (Fig. 2B).

**Table 2:** Number of patients with ocular toxicity by agent

**Agent**	**(n=1)**	**AKT inhibitor + FGFR inhibitor**	**(n=2)**	**Anti-PD-1 antibody**	**(n=2)**	**AURORA inhibitor**	**(n=1)**	**Axl/Mer inhibitor**	**(n=2)**	**CLK inhibitor**	**(n=2)**	**EP4 inhibitor**	**(n=14)**	FGFR inhibitor^*^	**(n=2)**	**HER2-directed ADC**	**(n=7)**	**HSP90 inhibitor**	**(n=1)**	**RET inhibitor**
Objective findings	Grade 1/2/3																			
– Conjunctivitis	—		—		—		—		1/0/0		—		—		—		—		—	
– Dim vision with abnormal ERG	—		—		—		—		—		—		—		—		1/1/0		—	
– Dry eye	—		—		—		1/0/0		1/0/0		—		1/1/0		—		—		—	
– Keratitis	—		0/1/0		—		—		—		—		—		—		—		—	
– Keratopathy	0/1/0		—		1/1/0		—		—		—		4/0/1		1/0/0		—		—	
– Retinopathy	—		1/0/0		—		—		—		2/0/0		8/2/0		—		1/0/0		0/1/0	
– Uveitis	—		—		—		—		—		—		—		0/1/0		—		—	
Subjective findings	Grade 1/2/3																			
– Dim vision	—		—		—		—		—		—		—		—		3/0/0		—	
– Flashing lights	—		—		—		—		—		—		—		—		0/0/1		—	
Temporary hold of drugs	1		2		0		0		1		1		10		1		5		1	
Recovered	1		2		2		1		2		2		16		1		6		1	
Days to recovery	70		13–42		56–196		14		12–32		30–62		7–156		26		32–464		36	

*ADC* Antibody–drug conjugate, *CLK* CDC-like kinase, *EP4* prostaglandin E2 receptor 4, *ERG* electroretinography, *FGFR* fibroblast growth factor receptor, *HER2* human epidermal growth factor receptor 2, *HSP90* heat shock protein 90, *PD-1* programmed death-1, *RET* rearranged during transfection

^*^Three patients had multiple symptoms

### Recovery from ocular toxicity

Drug administration was subjected to a temporary hold due to ocular toxicity in 22 cases. For patients with conjunctivitis, uveitis, and keratopathy, temporary hold of the drug was less frequent: 0 of the 1 patient with conjunctivitis, 0 of the 1 patient with uveitis, 4 of the 9 patients with keratopathy. Some patients were treated with eye drops and laser therapy. Except for three cases, all patients fully recovered from their ocular toxicities. Recovery was not confirmed in the three patients because the trial was stopped for two patients and one patient died; these patients experienced keratopathy due to an FGFR inhibitor, uveitis due to HER2-directed ADC, and dim vision (where patients see darker images) due to an HSP90 inhibitor (follow up time: 4–196 days) (Table 2 and Fig. 2A).

Among 37 ocular toxicities in 33 patients, 19 cases recovered to grade 0 within 50 days from onset, while the patient with dim vision due to an HSP90 inhibitor required more than 400 days to recover.

### Objective findings of ocular toxicity

**Fig. 3:**
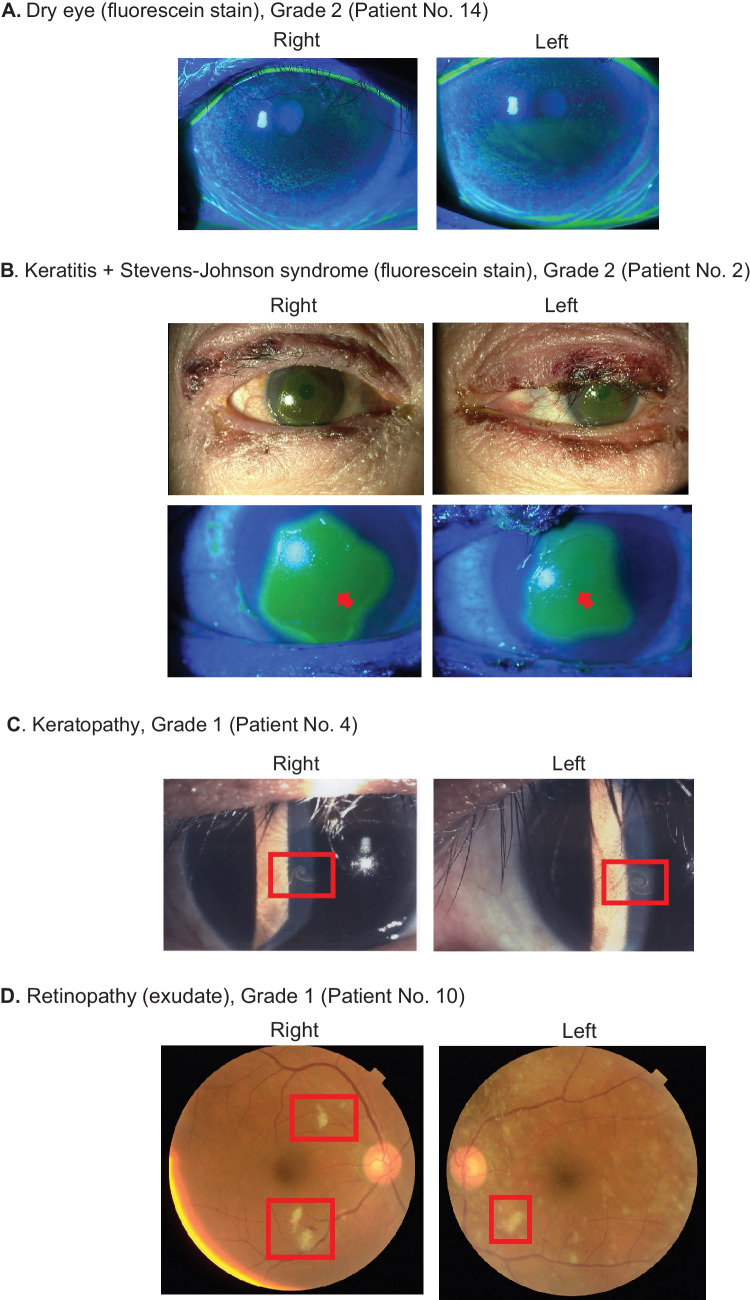

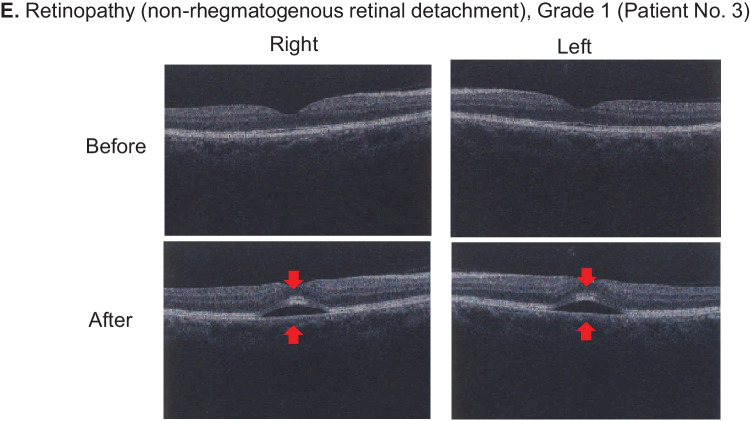
Representative objective findings of ocular toxicity. **A** Superficial punctate keratopathy of bilateral cornea with fluorescein staining. **B** Epithelial defects of bilateral cornea due to Stevens-Johnson syndrome. **C** Vortex keratopathy in bilateral cornea. **D** Multiple soft exudate in bilateral retina. **E** Non-rhegmatogenous retinal detachment with bilateral subretinal fluid collection

Except in one case, ocular toxicities occurred bilaterally. One patient treated with an Axl/Mer inhibitor experienced unilateral dry eye. Representative objective findings of ocular toxicity are shown in Fig. 3.

## Discussion

This study examined 37 ocular toxicities in 33 patients treated with investigational drugs in early phase clinical trials conducted in the NCCH. A high incidence of ocular toxicities was observed following treatment with HSP90 inhibitors and FGFR inhibitors. Some ocular toxicities arose after a long period of time from the initial administration of the drug. Most patients developed ocular toxicities even though their dose was below the MTD. Ocular toxicities were generally not severe and were reversible after a temporary hold of the drug. Bilateral ocular toxicity could be used as a trigger for diagnosis of drug-induced toxicities.

Four patients had unique ocular toxicities with only subjective symptoms, lacking objective findings. For example, patients with dim vision related to an HSP-90 inhibitor experienced subjective symptoms such as seeing darker images, as if the lights were off even in bright places. Not all patients showed electroretinography changes, probably because damage to the outer retinal layer related to the HSP-90 inhibitor was minimal. There were 12 patients without subjective symptoms and showing only objective findings. Their ocular toxicities were detected by objective findings prior to the onset of subjective symptoms. Hence, we were able to start the treatment early and to prevent toxicities from becoming serious. In future early phase clinical trials, ophthalmologic examinations should be carefully considered after toxicity studies in vivo.

Some differences between previous clinical trials in the types and frequencies of ocular toxicities were observed. While dry eye and uveitis are reportedly common ocular toxicities associated with ICIs [1], neither was observed in this study. Compared to ocular toxicities due to FGFR inhibitors being reported in 13–41% of patients [2, 4–6], we observed these more frequently (53.8%). These differences may be due to the limited number of patients included in this study, although racial differences may also have played a part.

The specific patient group, which only included those involved in early clinical trials, is a limitation. Further, the detailed mechanisms of the toxicities are still unknown. In the late phase trials with more patients joined, careful watching on the severity and the timing of onset of ocular toxicities, depending on each type of drug, is needed.

## Conclusions

This study summarized the various ocular toxicities and their features identified in early phase clinical trials of investigational drugs. The risks and timing of the onset varied among ocular toxicities of anti-cancer drugs, which were fundamentally controllable. Oncologists as well as ophthalmologists should increase their knowledge about typical ocular toxicities of anti-cancer drugs.

## Data Availability

The data that support the findings of this study are available from the corresponding author upon reasonable request.
